# Hyaluronan suppresses enhanced cathepsin K expression via activation of NF-κB with mechanical stress loading in a human chondrocytic HCS-2/8 cells

**DOI:** 10.1038/s41598-019-57073-8

**Published:** 2020-01-14

**Authors:** Mochihito Suzuki, Nobunori Takahashi, Yasumori Sobue, Yoshifumi Ohashi, Kenji Kishimoto, Kyosuke Hattori, Naoki Ishiguro, Toshihisa Kojima

**Affiliations:** Department of Orthopedic Surgery, Nagoya University Graduate School of Medicine, 65 Tsurumai-cho, Showa-ku, Nagoya 466-8550 Japan

**Keywords:** Molecular medicine, Osteoimmunology

## Abstract

Cathepsin K is a protease known to be involved in not only bone remodeling and resorption, but also articular cartilage degradation that leads to osteoarthritis (OA). Hyaluronan (HA) plays a pivotal role in maintaining homeostasis within articular chondrocytes. Intra-articular supplementation of high molecular weight hyaluronan (HMW-HA) has been widely used in OA treatment. However, its prospective mechanism of action is still unclear. In this study, we examined the suppressive effect of HA on enhanced cathepsin K expression induced by mechanical stress loading. A human chondrocytic HCS-2/8 cells were cultured in silicon chambers and subjected to cyclic tensile stress (CTS) loading. CTS loading significantly increased messenger ribonucleic acid and protein expression of cathepsin K, which appeared to be suppressed by pre-treatment with HMW-HA. Activation of nuclear factor-kappa B (NF-κB) was induced by CTS loading, and suppressed by pre-treatment with HMW-HA. Helenalin, a chemical inhibitor of NF-κB, clearly suppressed the enhanced expression of cathepsin K, as well as NF-κB activation induced by CTS loading. The suppressive effect of HMW-HA on enhanced cathepsin K expression via NF-κB inhibition impacts the effectiveness of HMW-HA in OA treatment. Our findings provide new evidence supporting the biological effectiveness of intra-articular HMW-HA injections for treatment of OA.

## Introduction

Osteoarthritis (OA) is a prevalent chronic joint disease associated with cartilage degeneration that tends to increase with age in modern society. This condition affects 240 million people globally, with 9.6% of men and 18% of women aged ≤60 years having symptomatic OA^1^. In clinical practice, OA associated with cartilage degeneration is encountered frequently. OA is associated with many risk factors, including age, obesity, genetic factors, and mechanical stress loading^2^. Excess mechanical stress loading is an important contributor to the development of OA, but the mechanisms through which it induces chondrocyte degeneration or cartilage degradation are unclear. Several previous studies have described the catabolic effects of mechanical stress loading in articular cartilage^3^. We previously reported that CD44, a primary receptor for hyaluronan (HA), was significantly cleaved and fragmented in articular chondrocytes obtained from human OA cartilage, with excess mechanical stress loading inducing CD44 cleavage via increased expression of a disintegrin and metalloprotease 10 (ADAM10)^4–6^. In this study, we used a mechanical stress loading system in a chondrocytic cell line mimicking chondrocyte degeneration in OA.

Intra-articular injection of high molecular weight hyaluronan (HMW-HA) has been frequently used in clinical practice as a treatment for OA since 1987^7–9^. HA plays an important role in maintaining articular cartilage through suppression of inflammation, pain relief, and improvement of endogenous HA production and properties of synovial fluid^10^. Although various mechanisms of action for HMW-HA, as well as its clinical effectiveness for treatment of OA, have been reported previously^7,10^, its prospective mechanism is not fully understood. In order to elucidate the molecular mechanisms of action of HMW-HA in articular cartilage degeneration, cathepsin K expression in chondrocytes needs to be examined.

Cathepsin K, a cysteine protease, is involved in the degradation of key components of bone and cartilage such as type I and type II collagen. This enzyme is known to be involved in bone remodeling/resorption and articular cartilage degradation^11,12^, and is reportedly expressed in articular chondrocytes other than osteoclasts and synovial fibroblasts^13^. The cleavage of type II collagen by cathepsin K is increased in human OA articular cartilage^14^. We previously reported that HMW-HA suppressed the increased cathepsin K expression induced by lipopolysaccharide (LPS) in human fibroblasts^15^. However, no reports have described changes in cathepsin K expression due to mechanical stress loading, or the effect of HMW-HA on cathepsin K expression in chondrocytes.

In this study, we examined changes in expression of cathepsin K induced by mechanical stress loading in a human chondrocytic cell line (HCS-2/8). We also explored the suppressive effect of HMW-HA on cathepsin K expression. Our findings provide new evidence supporting the biological effectiveness of intra-articular HA injections for the treatment of OA.

## Results

### Induction of cathepsin K expression by CTS loading

**Figure 1:**
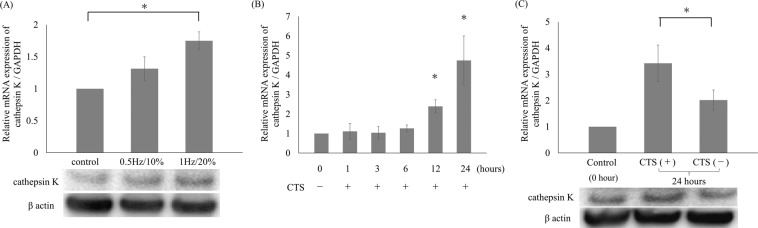
Induction of cathepsin K expression with CTS loading. Cathepsin K mRNA and protein expression levels. Different intensities of CTS loading were applied for 24 hours. Cathepsin K expression was significantly enhanced at higher intensities of CTS loading (1 Hz and 20% elongation; *p < 0.05). (**A**) Cathepsin K mRNA expression was time-dependently increased up to 24 hours under CTS loading at 1 Hz and 20% elongation with statistical significance at 12 and 24 hours, as compared to the untreated control (*p < 0.05). (**B**) Cathepsin K mRNA expression was increased with CTS loading at 1 Hz and 20% elongation for 24 hours, as compared to a sample cultured without CTS loading for 24 hours. Cathepsin K protein expression was also enhanced by CTS loading at the same intensity, as demonstrated in Western blotting analysis (*p < 0.05) (**C**).

HCS cells (2 × 10^5^ cells) were pre-cultured in 10 cm² silicon chambers (STB-CH-10, STREX, Japan) pre-coated with type I collagen (COL1) (Cellmatrix®, Nitta Gelatin, Japan) for two days. The cells in full confluence were stimulated with CTS loading using STB-140 (STREX) at various loading intensities, as follows: control without CTS loading; 30 cycles/min (0.5 Hz) and 10% elongation; and 60 cycles/min (1 Hz) and 20% elongation (Supplementary Fig. S1). The mRNA expression of cathepsin K was significantly increased with CTS loading at 1 Hz and 20% elongation for 24 hours, as compared to the untreated control (p < 0.05; Fig. 1A). To examine the time-dependency of cathepsin K mRNA expression, cells were subjected to CTS loading at 1 Hz and 20% elongation for 0, 1, 3, 6, 12, and 24 hours. Cathepsin K mRNA expression was significantly increased with CTS loading at 1 Hz and 20% elongation for 12 and 24 hours, as compared to 0 hours (p < 0.05; Fig. 1B). CTS loading appeared to significantly increase cathepsin K mRNA expression in both strength-dependent and time-dependent manners. The protein expression of cathepsin K was also increased with CTS loading at 1 Hz and 20% elongation for 24 hours (Fig. 1C).

### Inhibition of CTS loading-induced cathepsin K expression by HMW-HA

**Figure 2:**
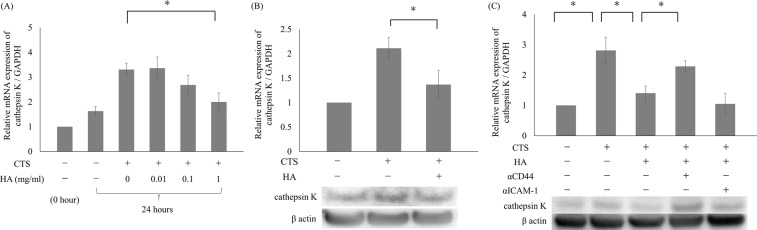
Suppressive effects of HMW-HA on enhanced cathepsin K expression induced by CTS loading at 1 Hz and 20% elongation for 24 hours. Pre-treatment with HA dose-dependently suppressed cathepsin K mRNA expression with statistical significance at a concentration of 1 mg/mL. (**A**) Pre-treatment with HA significantly suppressed the enhanced cathepsin K mRNA and protein expression induced by CTS loading for 24 hours, as compared to the sample without HA pre-treatment. Cathepsin K protein expression was also enhanced with CTS loading, as compared to the untreated sample cultured for 24 hours, as demonstrated by Western blotting analysis. (**B**) The suppressive effect of HA was cancelled by pre-treatment with anti-CD44 antibody for one hour, whereas no significant effect was observed with anti-ICAM-1 antibody pre-treatment (*p < 0.05) (**C**).

Cells were pre-treated with HMW-HA for one hour prior to CTS loading at 1 Hz and 20% elongation for 24 hours. Pre-treatment with 1 mg/mL HMW-HA significantly suppressed cathepsin K mRNA expression induced by CTS loading in a dose-dependent manner (p < 0.05; Fig. 2A). Pre-treatment with 1 mg/mL HMW-HA also suppressed the increased protein expression of cathepsin K induced by CTS loading for 24 hours (Fig. 2B).

To examine the primary HA receptor involved in the inhibitory effect of HMW-HA described above, cells were treated with anti-CD44 or anti-ICAM-1 antibodies for one hour before the one-hour pre-treatment with HMW-HA. As shown in Fig. 2C, treatment with anti-CD44 antibody clearly cancelled the inhibitory effect of HMW-HA. The difference between the samples pre-treated with HMW-HA with or without the anti-CD44 antibody treatment was statistically significant (p < 0.05). Conversely, treatment with anti-ICAM-1 antibody did not affect the inhibitory effect of HMW-HA on cathepsin K mRNA expression induced by CTS loading (P < 0.05). Western blotting analysis demonstrated similar results for changes in cathepsin K mRNA expression.

### HMW-HA suppressed NF-κB activation induced by CTS loading

**Figure 3:**
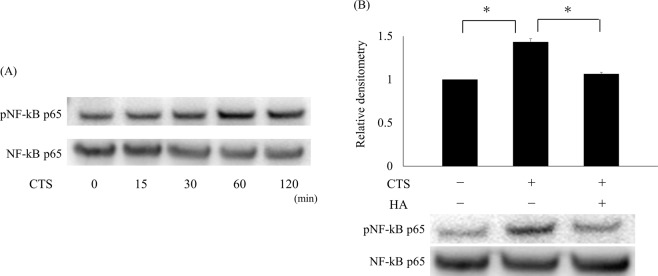
Effects of HMW-HA on enhanced activation of NF-κB induced by CTS loading. Phosphorylation of NF-κB p65 was time-dependently enhanced by CTS loading at 1 Hz and 20% elongation, as evaluated by Western blotting analysis. Phosphorylation reach was strongest at 60 minutes. (**A**) Pre-treatment with HA suppressed NF-κB p65 phosphorylation induced by CTS loading for 60 minutes, as compared to the sample without HA pre-treatment. Band densitometry demonstrated significant suppression of NF-κB p65 phosphorylation with HA pre-treatment. The band density of phospho-NF-κB p65 was enhanced in samples without HA pre-treatment (*p < 0.05) (**B**).

Cells were stretched with CTS loading at 1 Hz and 20% elongation for 120 minutes, and NF-κB activation was examined at 0, 15, 30, 60, and 120 minutes. Phosphorylation of NF-κB p65 was clearly induced after 60 minutes of CTS loading, as evaluated by Western blotting analysis (Fig. 3A). Cells were then pre-treated with HMW-HA for one hour and subjected to 60 minutes of CTS loading. Pre-treatment with HMW-HA significantly suppressed the phosphorylation of NF-κB p65 induced by CTS loading, as evaluated by Western blotting and band densitometry (p < 0.05; Fig. 3B).

### NF-kB immunofluorescence staining in cells stimulated with chemical TRPV-4 agonist mimicking the signal transduction induced by CTS loading

**Figure 4:**
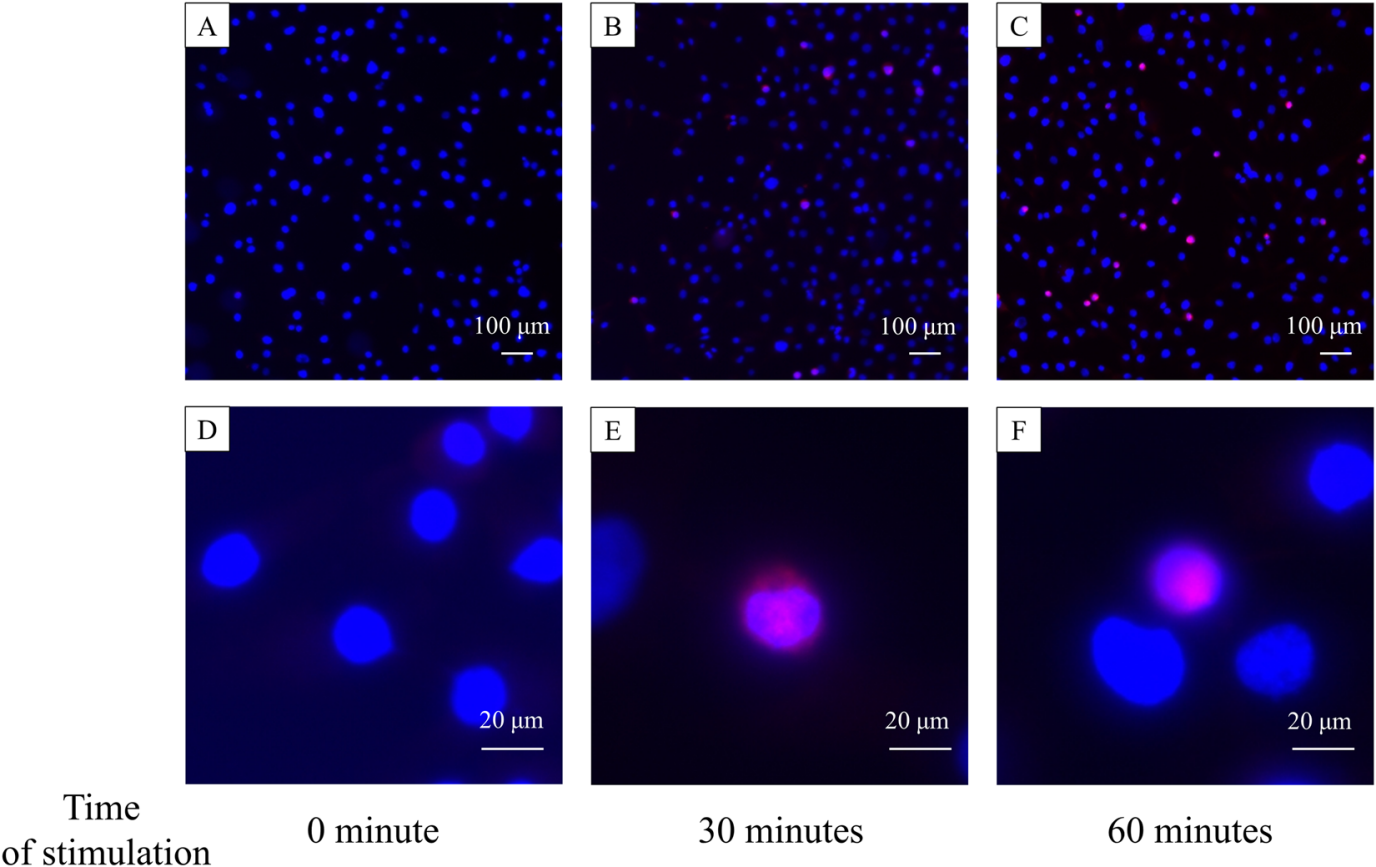
Immunofluorescence staining of HCS cells using anti-phospho-NF-kB p65 antibody. The HCS cells were stimulated with GSK1016790, a transient receptor potential vanilloid 4 (TRPV4) agonist, for 30 or 60 minutes. The chemical activation of TRPV-4 mimicked the signal transduction induced by CTS loading. There observed only nuclear staining (blue) in the untreated control samples. (**A**,**D**) The major stained region with anti-phospho-NF-kB p65 antibody (red) was cytoplasm at 30 minutes (**B**,**E**) and nuclei at 60 minutes (**C**,**F**).

In order to confirm the NF-kB activation and nuclear translocation, we performed the immunofluorescence staining using anti-phospho-NF-kB p65 antibody. We could not use the cells cultured on silicone chambers for immunofluorescence staining since they are unsuitable for the observation with our fluorescence microscope. We cultured the HCS cells on chamber slides for immunofluorescence staining this time. However, we cannot apply the CTS loading to cells cultured on hard glass slides. Then, we decided to stimulate cells chemically with GSK1016790 (GSK), a transient receptor potential vanilloid 4 (TRPV4)-agonist. TRPV4, a member of the vanilloid subfamily of the transient receptor potential (TRP) superfamily of ion channels, was shown to function as a primary mechanoreceptor in articular chondrocytes^16^. To determine the sufficient concentration of GSK to activate NF-kB, HCS cells cultured on 6 wells plate (2 × 10^5^ cells/well) were stimulated with various concentration of GSK for 24 hours. The mRNA and protein expression of cathepsin K was increased with the GSK stimulation in a dose-dependent manner with statistical significance at ≥1000 nM (p < 0.01) (Fig. S2). To confirm the activation of NF-kB by western blotting analysis, cells were stimulated with 1000 nM GSK and phosphorylation of NF-κB p65 was examined at 0, 30, 60, and 120 minutes. Phosphorylation of NF-κB p65 was clearly induced at 60 minutes of GSK stimulation with statistical significance (P < 0.05) (Fig. S3). HCS cells cultured on 2 chamber slides (1 × 10^5^ cells/well) were stimulated with 1000 nM GSK for 30 or 60 minutes. We observed only the nuclear staining (blue) in the untreated control cells (Fig. 4A,D). The major stained region with anti-phospho-NF-kB p65 antibody (red) was cytoplasm at 30 minutes (Fig. 4B,E) and nuclei at 60 minutes (Fig. 4C,F). The phosphorylation and nuclear translocation of NF-kB was clearly observed with the chemical TRPV-4 activation mimicking the signal transduction induced by CTS loading.

### Chemical inhibition of NF-κB suppressed cathepsin K expression induced by CTS loading

**Figure 5:**
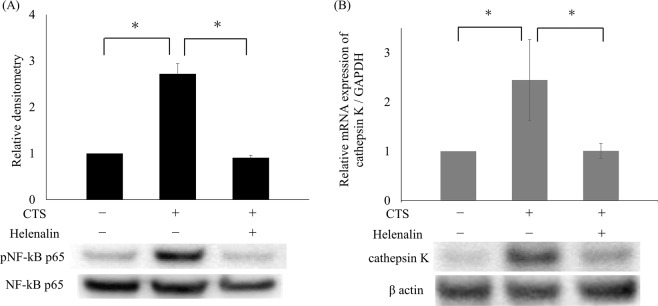
Effects of helenalin, a specific NF-κB inhibitor, on NF-κB activation and cathepsin K expression induced by CTS loading (1 Hz and 20%). Helenalin pre-treatment suppressed the phosphorylation of NF-κB p65 induced by CTS loading for 60 minutes. Densitometry demonstrated significant suppression of NF-κB p65 phosphorylation in samples pre-treated with helenalin, as compared to those without pre-treatment. (**A**) Helenalin pre-treatment significantly suppressed the enhanced cathepsin K mRNA and protein expression induced by CTS loading for 24 hours (*p < 0.05) (**B**).

To investigate whether NF-κB activation was responsible for the significant increase in cathepsin K expression induced by CTS loading, cells were pretreated with 1 μM helenalin, a specific NF-κB inhibitor, for one hour prior to CTS loading for 60 minutes. Helenalin pre-treatment significantly suppressed the increased phosphorylation of NF-κB p65 induced by CTS loading, as evaluated by Western blotting and densitometry (p < 0.05; Fig. 5A). Pre-treatment with 1 μM helenalin also significantly suppressed the increased expression of cathepsin K mRNA (p < 0.05) and protein induced by 24 hours of CTS loading (Figs. 5B and S4).

### Induction of cathepsin K expression and suppressive effect of HMW-HA in three-dimensional environment

**Figure 6:**
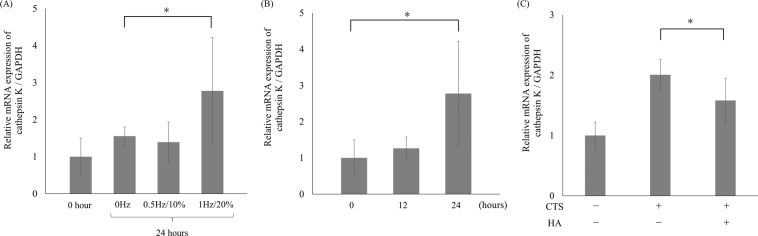
Induction of cathepsin K expression by CTS loading and suppressive effect of high molecular-weight (HMW)-HA in HCS cells cultured in three-dimensional environment. Different intensities of CTS loading were applied to the cells embedded in type 1 collagen gels for 24 hours. Cathepsin K expression was significantly enhanced by higher intensities of CTS loading (1 Hz and 20% elongation), as compared to the untreated control (*p < 0.05). (**A**) Cathepsin K mRNA expression was time-dependently increased with CTS loading at intensity of 1 Hz and 20% elongation with statistical significance at 24 hours, as compared to the untreated control (*p < 0.05). (**B**) Pre-treatment with 1 mg/mL HMW-HA significantly suppressed the enhanced cathepsin K mRNA expression induced by CTS loading for 24 hours, as compared to the sample without HMW-HA pre-treatment (*p < 0.05) (**C**).

We examined change in cathepsin K mRNA expression using three-dimensional cell culture system. The HCS cells embedded in COL1 gel (5 × 10^5^ cells) were pre-cultured for two days followed with the CTS loading at various loading intensities. The mRNA expression of cathepsin K was significantly increased with CTS loading at intensity of 1 Hz and 20% elongation for 24 hours as compared to the untreated control (Fig. 6A). To examine the time-dependency of cathepsin K mRNA expression, cells were subjected to CTS loading at 1 Hz and 20% elongation for 0, 12, and 24 hours. Cathepsin K mRNA expression was significantly increased with 24 hours of CTS loading as compared to baseline (0 hour) (Fig. 6B). To examine the effect of HMW-HA, cells were pre-treated with 1 mg/mL HMW-HA for one hour followed with 24 hours of CTS loading at 1 Hz and 20% elongation. Pre-treatment with HMW-HA significantly suppressed the cathepsin K mRNA expression induced by CTS loading (Fig. 6C). These data obtained by using the 3D culture system were quite similar to those using the monolayer culture system.

### Induction of cathepsin K expression and suppressive effect of HMW-HA in primary articular chondrocytes

**Figure 7:**
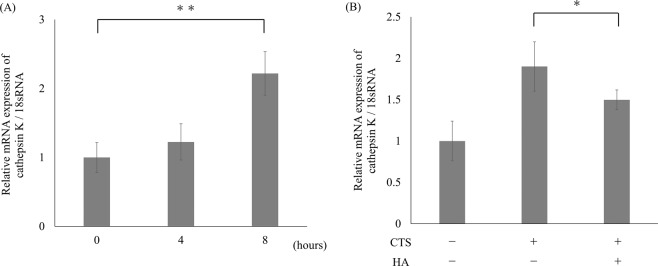
Induction of cathepsin K expression by CTS loading and suppressive effect of high molecular-weight (HMW)-HA in primary bovine articular chondrocytes. The CTS loading increased the cathepsin K mRNA expression levels at intensity of 0.5 Hz and 10% elongation in a time-dependent manner with statistical significance at 8 hours (**p < 0.01). (**A**) Pre-treatment with 1 mg/mL HMW-HA significantly suppressed the enhanced cathepsin K mRNA expression induced by 8 hours of CTS loading (*p < 0.05) (**B**).

We examined the effect of CTS loading on the mRNA expression of cathepsin K using primary articular chondrocytes. Primary bovine articular chondrocytes (BACs) were pre-cultured on 10 cm² silicon chambers coated with COL1 for two days. The BACs in full confluence were subjected to 0, 4, and 8 hours of CTS loading at intensity of 0.5 Hz and 10% elongation by using STB-140. The CTS loading increased the cathepsin K mRNA expression in a time-dependent manner (Fig. 7A). To examine the suppressive effect of HMW-HA on the enhanced cathepsin K expression, BACs were pre-treated with 1 mg/mL HMW-HA for one hour followed with the 8 hours of CTS loading at intensity of 0.5 Hz and 10% elongation. Pre-treatment with HMW-HA significantly suppressed the cathepsin K mRNA expression induced by CTS loading (p < 0.05; Fig. 7B).

## Discussions

In this study, we found that CTS loading significantly increased the expression of cathepsin K via activation of the NF-κB signal pathway in both strength-dependent and time-dependent manners in a human chondrocytic cell line. We also found that pre-treatment with HMW-HA decreased the increased cathepsin K expression via suppression of NF-κB activation.

Cathepsin K, a lysosomal cysteine proteinase, is involved in the cleavage of type II collagen in OA^14^. Previous studies have reported an increased expression of cathepsin K in human OA cartilage and chondrocytes, as compared to healthy cartilage and chondrocytes^13^. They also demonstrated that OA progression was delayed in cathepsin K knockout mice using a surgical OA model^17,18^. Several other studies have reported that inhibition of cathepsin K resulted in reduced cartilage degeneration and inflammation in mouse OA models and collagen-induced arthritis^19–21^. Based on these previous studies, cathepsin K is a potential treatment target in OA. Thus, the suppression of enhanced cathepsin K expression induced by excess mechanical stress loading might be the molecular mechanism underlying the clinical effectiveness of intra-articular HA injections.

We observed the significant increasing of cathepsin K at intensity of 1 Hz and 20% elongation in this study. Excess mechanical stress loading has been reported to induce the catabolic change in chondrocytes. Catabolic genes (MMP-1, MMP-3, MMP-13 etc.) were reported to be upregulated at various protocols with a frequency of 0.5 Hz and strain magnitudes of between 7 and 23% for 3 to 48 hours depending on cell types^3^. Thus, it is necessary to verify the sufficient intensity of mechanical stress loading to induce the catabolic change for each cell type. We observed almost no difference in cathepsin K expression at intensity of 0.5 Hz and 10% elongation in HCS in the current study. This experimental condition would be the physiological intensity of mechanical stress loading and the intensity of 1 Hz and 20% elongation is, so called, the excess mechanical stress loading in HCS.

HA plays an important role in maintaining homeostasis of articular cartilage. Exogenous HA has been reported to demonstrate anti-inflammatory activity via association with its receptors^10^. HA is known to associate with several surface molecules; CD44 and ICAM-1 are the best-known HA receptors^22^. Different HA receptors are reportedly responsible for different situations. We reported previously that CD44 was mainly involved in the suppressive effect of HA on enhanced cathepsin K expression induced by LPS stimulation, whereas ICAM-1 was involved in matrix metalloproteinase-1 (MMP-1) expression in human fibroblasts^15^. Ozawa *et al*. reported that both CD44 and ICAM-1 were partially involved in the suppressive effect of HA on enhanced ADAMTS-4, 5, and MMP-13 expression induced by CTS loading^23^. In the present study, CD44, but not ICAM-1, was involved in the suppressive effect of HA on enhanced cathepsin K expression induced by mechanical stress loading. Multiple receptors involved in association with HA may explain the various bioactivity and clinical effects demonstrated by HA.

Transcription of NF-κB plays a critical role in the induction of catabolic enzymes, such as ADAMTSs and MMPs^23–25^. The NF-κB pathway is reportedly activated by mechanical stress loading^26,27^, and CTS loading has been shown to induce NF-κB activation in human chondrocytes^23^. Our study also demonstrated that CTS loading induced NF-κB activation, leading to cathepsin K expression, in a human chondrocytic cell line. In addition, inhibition of NF-κB by HA or helenalin effectively suppressed cathepsin K expression. Inhibition of the NF-κB pathway may be a part of the molecular mechanism of action of HA in the treatment of OA.

This study has some limitations. First, the stretching device we used cannot provide the compressive force which should be experienced by chondrocytes *in vivo*. However, many previous reports have demonstrated the effect of CTS on protease expression change^3^. We think the stretching system could give us at least fragmentary knowledge of effect of mechanical stress on chondrocytes. Future studies should examine the different types of mechanical stress, such as compression, shear forces, and hydrostatic pressure.

Excess mechanical stress loading induced the expression of cathepsin K in a human chondrocytic cell line HCS-2/8. Activation of NF-κB was strongly involved in this induction; pre-treatment with HMW-HA effectively suppressed both NF-κB activation and the resulting cathepsin K expression under mechanical stress loading. These results may explain the mechanism behind the catabolic effects of excess mechanical stress loading, which lead to OA, and also provide new evidence supporting the biological effectiveness of intra-articular HA injections for the treatment of OA.

## Materials and Methods

### Reagents

HMW-HA (Artz®, average 900 kDa) was purchased from Seikagaku Corporation (Tokyo, Japan). Rabbit anti-cathepsin K (clone EPR19992) monoclonal antibody (ab207086), rat anti-CD44 (clone Hermes-1) monoclonal antibody (ab119335), mouse anti-intercellular adhesion molecule-1 (anti-ICAM-1; clone 1A29) monoclonal antibody (ab171123), and helenalin (an inhibitor of nuclear factor-kappa B [NF-κB]; ab146197) were purchased from Abcam (Tokyo, Japan). Rabbit anti-human NF-κB p65 (#8242), phospho-NF-κB p65 (#3033), beta actin antibody (#4970), and horseradish peroxidase (HRP)-conjugated goat anti-rabbit (#7074) IgG were purchased from Cell Signaling Technology (Beverly, MA, USA). Dulbecco’s modified Eagle’s medium (DMEM) and trypsin-ethylenediaminetetraacetic acid (trypsin-EDTA), and GSK1016790 (ab146191) were obtained from Sigma-Aldrich Co. (St. Louis, MO, USA). Fetal bovine serum (FBS) was purchased from PAA Laboratories GmbH (Pasching, Austria).

### Cells and culture

HCS-2/8, a human chondrocytic cell line, is a continuous long-term culture cell line derived from a human chondrosarcoma^28^. In monolayer cultures, HCS cells (2 × 10^5^ cells) were cultured in 10 cm² silicon culture chambers (STB-CH-10, STREX, Japan) precoated with 10% type I collagen (COL1) (Cellmatrix®, Nitta Gelatin, Japan) in DMEM with 5% FBS at 37 °C in a 5% CO_2_ atmosphere. After incubation for 48 hours, cells were washed twice with phosphate-buffered saline (PBS), and then starved overnight in serum-free conditions. After the overnight starvation, cells were pre-treated with 1 mg/mL HA for one hour, subjected to cyclic tensile stress (CTS) loading using the automated cell stretching system STB-140 (STREX, Japan), and harvested. Levels of messenger ribonucleic acid (mRNA) and protein expression were measured by real-time polymerase chain reaction (PCR) and Western blotting analysis, respectively. In three-dimensional (3D) cultures, cells were embedded in COL1 gel. The Cellmatrix I-A COL1 gel (Nitta Gelatin, Osaka, Japan), comprising 0.3% COL1 from porcine tendons, was used as a scaffold. Collagen solution, 5 × DMEM (including cells) and buffer (comprising 100 ml of 0.05 N NaOH solution, 2.2 g NaHCO_3_ and 200 mM HEPES) were mixed at a ratio of 7:2:1. A2-ml sample of the mixture was then poured into the post chamber, incubated at 37 ◦C and allowed to polymerize. Two milliliters culture medium was overlaid. The final cell concentration was 5 × 10^5^ cells/chamber.

In experiments using primary cells, bovine articular chondrocytes (BACs) were isolated from full thickness slices of the articular surface of metacarpophalangeal joints of young adult steers (aged 18–24 months), which were provided by Nagoya City Central Wholesale Market. These slices were digested in 0.2% (0.05 g) pronase (catalogue number: 537088, activity: ≥70,000 proteolytic units/g dry weight, Merck, Germany) for 1 hour at 37 °C and subsequently in 0.025% (0.00625 g) collagenase P (catalogue number: 11213865001, activity: >1.5 U/mg lyophilizate, Roche, Germany) overnight at 37 °C^4,29^. Cells were cultured in DMEM/Ham’s F12 medium with 1 × insulin-transferrin-sodium selenite (ITS), 4% FBS, 100 units/ml penicillin, 100 μg/ml streptomycin, and 0.25 μg/ml amphotericin at 37 °C in a 5% CO_2_ environment. The presence of ITS maintains the chondrocyte phenotype^30,31^. For CTS loading experiments, 1 × 10^6^ cells were cultured on 10 cm^2^ dedicated silicon culture chambers, which were coated with type 1 collagen. After incubation for 48 hours in 4% FBS, cells were washed twice with PBS, and then starved overnight in serum-free conditions. Subsequently, cells were stimulated using the automated cell stretching system STB-140 under serum-free conditions^5^.

### Real-time PCR

Total ribonucleic acid (RNA) was extracted using the RNeasy Mini Kit (Qiagen, Germany) according to the manufacturer’s instructions. Reverse transcription was performed with the High Capacity cDNA Reverse Transcription Kit (Applied Biosystems, CA, USA) at 37 °C for 120 minutes. Real-time PCR was carried out using the LightCycler System with FastStart Master SYBR Green I PLUS (Roche, USA). Primers for cathepsin K and glyceraldehyde-3-phosphate dehydrogenase (GAPDH) were all designed and synthesized by Sigma Aldrich. The following primers were used: cathepsin K (human), forward primer 5′-TTCCCGCAGAATGACAC-3′, reverse primer 5′-CTGGGGACTCAGATTTAAGA-3′; GAPDH (human), forward primer 5′-TGAACGGGAAGCTCACTGG-3′, reverse primer 5′-TCCACCACCCTGTTGCTGTA -3′, cathepsin K (bovine), forward primer 5′-ATGCCTATCAACAGGATGTGGG-3′, reverse primer 5′-CTCCTCAGGGTATAGAGCAAAGC-3′; 18sRNA (bovine), forward primer 5′-GTAACCCGTTGAACCCCATT-3′, reverse primer 5′-CCATCCAATCGGTAGTAGCG-3′. PCR conditions were as follows: two minutes at 50 °C and 10 minutes at 95 °C, followed by 40 cycles of 15 seconds at 95 °C and one minute at 60 °C. Data were collected in the last 30 seconds. Real-time PCR efficiencies and fold increases in mRNA copy numbers were calculated as previously described^32^.

### Western blotting

Total protein was extracted using Cell Lysis Buffer (#9803, Cell Signaling Technology, USA) containing a protease/phosphatase inhibitor cocktail (#5872, Cell Signaling Technology, USA). Protein samples were centrifuged at 300 g for 10 minutes at 4 °C. After centrifugation, supernatants were mixed with Lane Marker Reducing Sample Buffer (Thermo Fisher Scientific) and heated at 70 °C for 10 minutes. Samples were loaded and separated on NuPAGE Bis-Tris Mini Gels (Invitrogen, USA) under reducing conditions. Samples were then transferred onto a nitrocellulose membrane and blocked in 5% nonfat dry milk in Tris-buffered saline with 0.1% Tween 20 (TBS-T). Cathepsin K, β-actin, NF-κB p65, and phospho-NF-κB p65 were detected with primary antibodies, followed by the appropriate secondary antibodies. Detection was performed using chemiluminescence (Thermo, USA). Band intensities were captured with a digital image scanner and quantified using densitometry software (CS Analyzer 3.0; ATTO, Tokyo, Japan).

### Immunofluorescence staining

HCS cells (1 × 10^5^ cells) were cultured in 2 chamber slides (IWAKI SCIENCE PRODUCTS Dept., Japan) at 37 °C in a 5% CO_2_ atmosphere. After incubation for 48 hours, cells were washed twice with phosphate-buffered saline (PBS) and starved overnight in serum-free conditions. After starvation, cells were stimulated with 1000 nM GSK1016790 for 30 or 60 minutes. After stimulation, cells were fixed with 4% Paraformaldehyde for 15 minutes and blocked with 1% Bovine Serum Albumin (BSA)-PBS for 1 hour at room temperature. Cells were incubated with a primary antibody (anti-phospho-NF-κB p65) overnight at 4°C and with a secondary antibody (Cy^TM^3-conjugated AffiniPure Donkey Anti-Rabbit IgG (H + L), Jackson ImmunoResearch) for 1 hour at room temperature. Nuclear staining was performed using Hoeches (H3570, Thermo Fisher Scientific). Stained cells were observed with a fluorescence microscope BZ-X700 (KEYENCE, Japan).

### Statistical analysis

All experiments were repeated at least three times, and similar results were obtained. The Kruskal-Wallis test was used for multiple-group comparisons, and the Holm post hoc test was used to evaluate the significance of individual differences in two-group comparisons only if the Kruskal-Wallis test indicated significance. P-values less than 0.05 were considered statistically significant. All statistical analyses were performed with SPSS version 24.0.0 software (IBM Corp, NY, USA).

## Supplementary information

Supplementary Information 

## Data Availability

All data generated or analyzed during this study are included in this published article.
