# Supplementary material for: Hyaluronan suppresses enhanced cathepsin K expression via activation of NF-κB with mechanical stress loading in a human chondrocytic HCS-2/8 cells
Source: Sci Rep. 2020 Jan 14;10:216. doi: 10.1038/s41598-019-57073-8 (PMC6959248; doi:10.1038/s41598-019-57073-8)
Supplement: Supplementary file 1 — Supplementary Information [file 41598_2019_57073_MOESM1_ESM.pdf]

Hyaluronan suppresses enhanced cathepsin K expression via activation of NF-κB with mechanical stress loading in a human chondrocytic HCS-2/8 cells

Mochihito Suzuki, Nobunori Takahashi, Yasumori Sobue, Yoshifumi Ohashi, Kenji Kishimoto, Kyosuke Hattori, Naoki Ishiguro, and Toshihisa Kojima

Supplementary Figure S1.

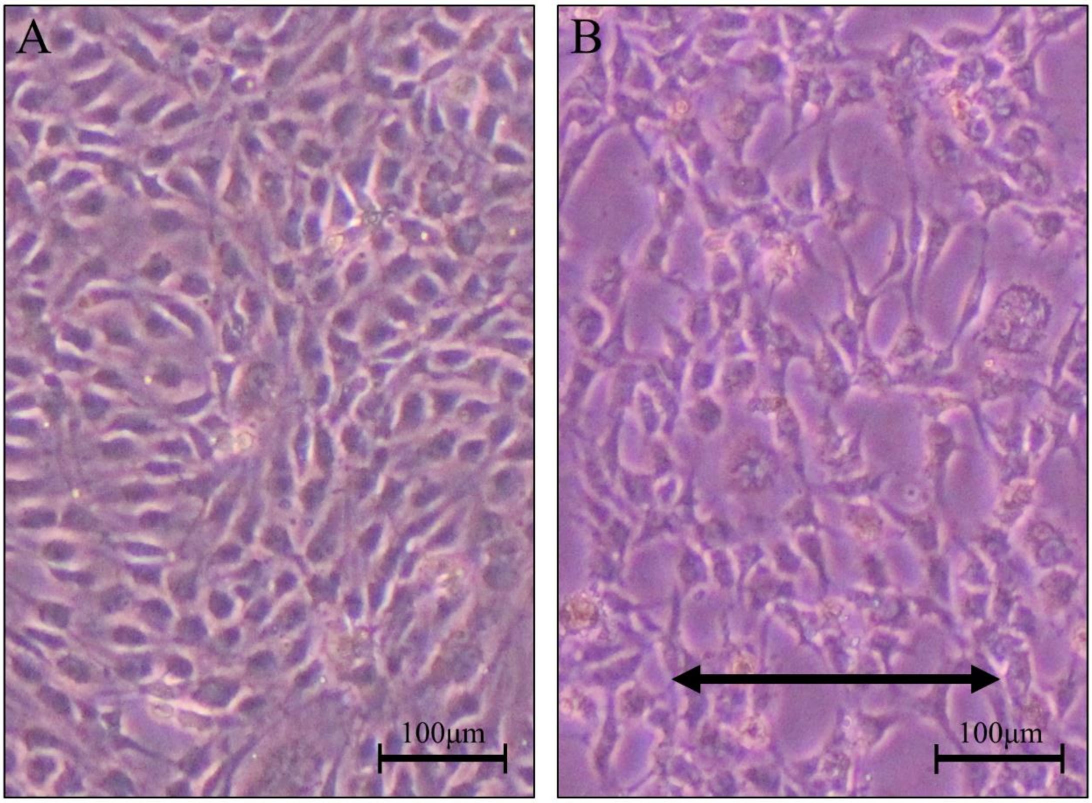

Supplementary Figure S2.

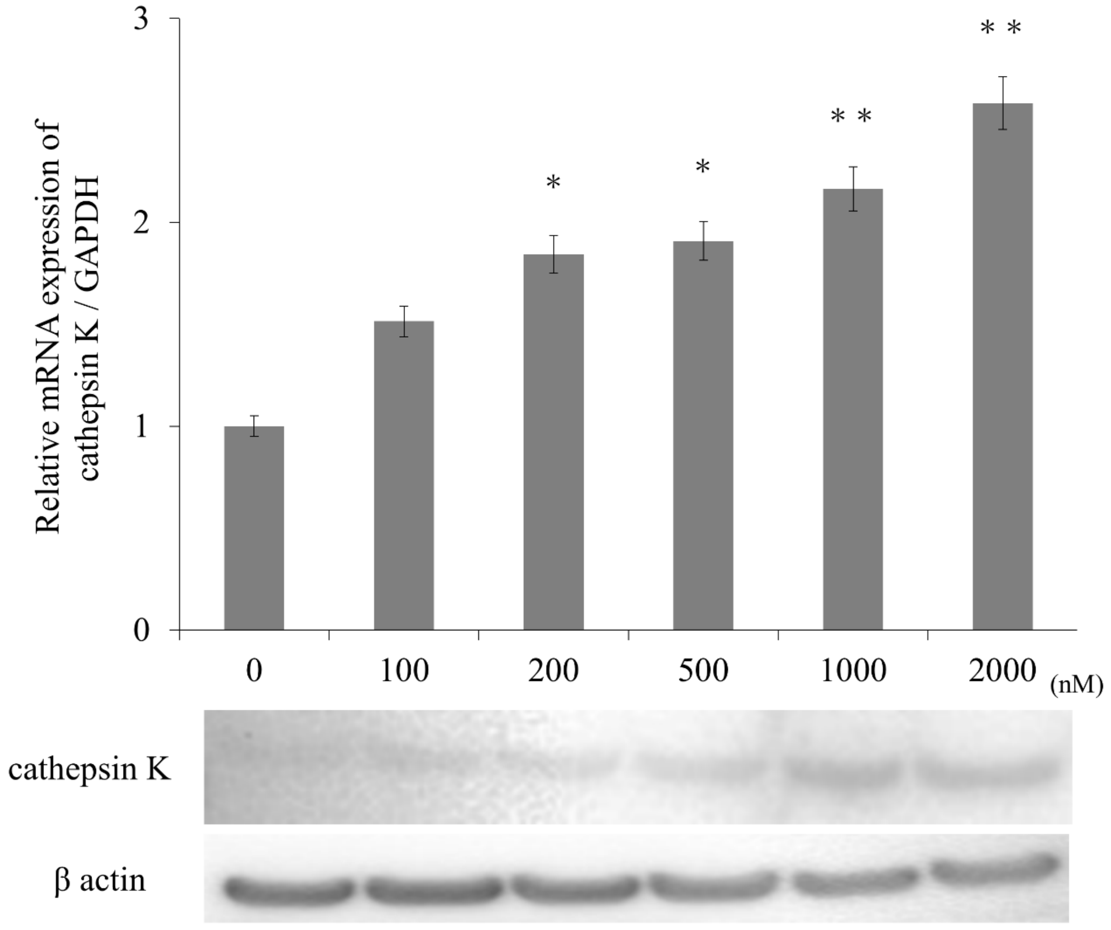

Supplementary Figure S3.

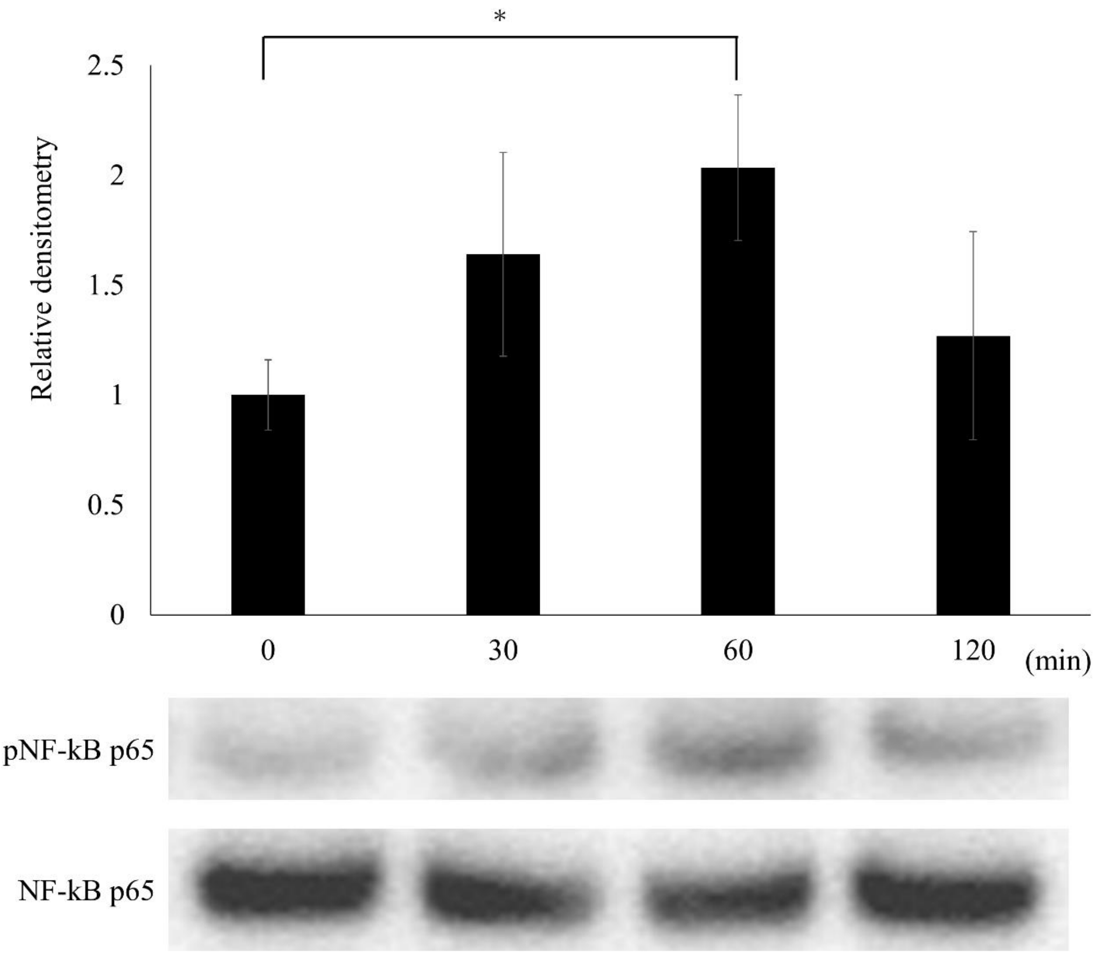

Supplementary Figure S4.

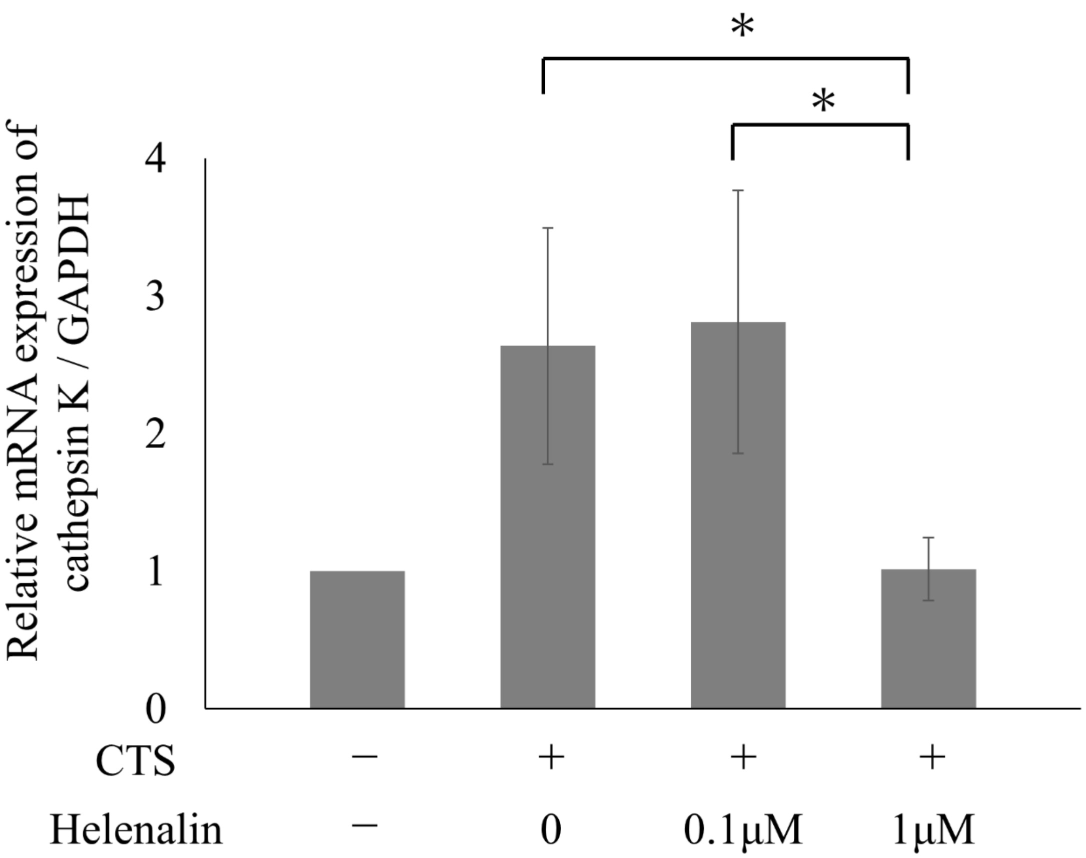

Supplementary Figure legends

Supplementary Figure S1. Phase contrast images of HCS in response to CTS loading. (A) Without stretch, HCS cultured on a silicon chamber for 24 hours. (B) HCS were stimulated with CTS loading at 1Hz and 20% elongation for 24 hours. Black double arrow on B shows stretch way. Black scale bar in bottom right on both figures indicates 100μm.

Supplementary Figure S2. Effect of transient receptor potential vanilloid 4 (TRPV4) activation on cathepsin K expression. Chemical stimulation with GSK1016790 (GSK), a TRPV4 agonist, for 24 hours increased the mRNA and protein expression of cathepsin K in a dose-dependent manner with statistical significance at ≥1000nM ($p<0.01$).

*p<0.05, **p<0.01

Supplementary Figure S3. Effects of chemical stimulation of transient receptor potential vanilloid 4 (TRPV4) on NF-κB activation. Phosphorylation of NF-κB p65 was time-dependently enhanced by chemical stimulation with GSK1016790 (GSK), a TRPV4 agonist, as evaluated by western blotting analysis. Phosphorylation enhancement reach statistical significance at 60 minutes (*p<0.05).

Supplementary Figure S4. Effects of helenalin at several dose on cathepsin K expression induced by CTS loading. Helenalin pre-treatment dose-dependently suppressed the enhanced cathepsin K mRNA expression induced by CTS loading for 24 hours (*$p<0.05$).
